# Morphology, Mechanical and Thermal Properties of Thermoplastic Polyurethane Containing Reduced Graphene Oxide and Graphene Nanoplatelets

**DOI:** 10.3390/ma11010082

**Published:** 2018-01-06

**Authors:** Michał Strankowski, Piotr Korzeniewski, Justyna Strankowska, Anu A. S., Sabu Thomas

**Affiliations:** 1 Gdansk University of Technology, Chemical Faculty, Polymer Technology Department, Narutowicza 11/12, 80-233 Gdansk, Poland; piotr.korzeniewski90@gmail.com; 2 Institute of Experimental Physics, Faculty of Mathematics, Physics and Informatics, University of Gdansk, Wita Stwosza 57, 80-308 Gdansk, Poland; fizjkr@ug.edu.pl; 3 International and Inter University Centre for Nanoscience and Nanotechnology, Mahatma Gandhi University, Kottayam, Kerala 686560, India; anumgu@gmail.com (A.A.S.); sabuthomas@mgu.ac.in (S.T.)

**Keywords:** polyurethane, nanocomposites, graphene, thermal analysis, mechanical properties

## Abstract

Polyurethane/graphene nanocomposites were synthesized using commercial thermoplastic polyurethane (TPU, Apilon 52DE55), and two types of graphene derivatives: graphene nanoplatelets (GNP) and reduced graphene oxide (RGO). Fourier Transformation Infrared Spectroscopy Fourier Transformation Infrared Spectroscopy (FTIR) spectroscopy, TEM, and SEM microscopy and XRD techniques were used to chemically and structurally characterize GNP and RGO nanofillers. The properties of the new TPU nanocomposite materials were studied using thermal analysis techniques (Dynamical Mechanical Analysis (DMA), Differential Scanning Calorimetry (DSC), Thermogravimetric Analysis (TG)) to describe the influence of graphene nanofillers on polyurethane matrix. Our investigation describes the comparison of two types of graphene derivatives, commercial one (GNP) and synthesized (RGO) on thermoplastic polyurethanes. These nanofillers provides opportunities to achieve compatibility with the TPU matrix. The property enhancements are attributed commonly to high aspect ratio of graphene nanoplatelets and filler–polymer interactions at the interface. The obtained nanocomposites exhibit higher thermal and mechanical properties due to the good dispersion of both nanofillers into TPU matrix. It was found that the addition of 2 wt % of the nanofiller could lead to a significant reinforcement effect on the TPU matrix. Also, with high content of nanofiller (GNP and RGO), the Payne effect was observed.

## 1. Introduction

Since the first scientific on the mechanical exfoliation of graphite into graphene monolayers by scotch-tape, several interesting studies have been published on graphene [1,2,3]. Graphene has become very popular research material during the last few years [2]. This method (scotch-tape approach) of obtaining graphene is inefficient for large-scale production [4]; therefore, many efforts have been undertaken to find a better way to get graphene monolayers. Promising large-scale methods of graphene fabricate are bottom-up methods (especially epitaxially growth on SiC crystal, chemical vapour deposition (CVD)), top-down methods, such as chemical, electrochemical, plasma-assisted, and mechanical exfoliation [5]. These methods mainly differ in the quality and efficiency of the obtained graphenes.

Bottom-up methods allow for the production of wafer graphene, which covers the demand of the electronic industry. The high quality of graphene monolayer shows the high electrical conductivity and transparency of this material. Quality of graphene produced by bottom-up approach shows well-builded one-layer structure without atomic defects [6]. The method of manufacturing this type of graphene was developed and patented by Institute of Electronic Materials Technology in Warsaw, Poland [7], and it is process of implementing at the industry scale. The main applications of these graphenes are focused to apply in displays, solar cells [8], integrated circuits elements (transistors [9]), and many other electronic devices.

Top-down approach of obtaining graphene is based on graphite as a raw material. In this way, graphite is exfoliated as the result of breaking inter-plane π-π interactions of stacked graphene in graphite crystal. Exfoliation can be carried in a mechanical, chemical, electrochemical, and plasma assisted way. According to top-down production methods, the obtained graphenes can differ in chemical and physical properties. The main differences are in crystal defects, various multi-layer stacked structure, polydispersity of basal plane, and thickness. The monolayer structure of top-down graphene can be folded and sharp-edged. We can also add function groups by various chemical modifications. These modifications have a strong effect on the electrical, mechanical, and optical properties of graphene obtained by top-down ways.

Top-down graphene is promising nanofiller for segmented polyurethane matrix. It can be introduced to matrix via in-situ polymerization, solvent-methods and during extrusion [10,11,12], or injection molding [8]. The addition of graphene allows for meeting the increasing requirements put in many applications. Besides the improvement of mechanical properties [10,11,12,13,14,15,16,17,18,19,20,21,22,23,24,25,26] and thermal stability [10,16,27,28], the modification of segmented polyurethanes by graphene can improve shape memory effect [15,21,24,25,29,30,31], gas-barrier properties [19], and electrical conductivity [16,18,19,23,32]. Graphene-polyurethane nanocomposites can be used as a selective membrane in filtration [33], piezoelectric sensors [34,35], actuators [30,36], or self-healing materials [20,37,38].

Elastomeric materials can be successfully modified with many nanofillers [39], especially clays, CNT [40], or graphene derivatives [41,42]. These different nano-modificators could cause high improvement in the properties (at low addition, up to 5%) of the polyurethane (PU) matrix because of the interactions between polyurethane chains and fillers at the nano-scale levels. Also, specific geometry (one-dimensional (1D), two-dimensional (2D), and three-dimensional (3D)) of these nanofillers [43] influence on the mechanical [44], thermal [44], or barrier properties [19] of polyurethane nanocomposites. Simply unmodified nanofillers have limited use, because of the compatibility issues of the polyurethane chains of hard and soft segments with the nano scale fillers. Therefore, the useful list of nanofillers include some “defected” form of graphene, modified CNT, and organically modified clays. The most important improvement of polyurethane matrix was achieved by using OMMT, CNT, or graphene derivatives, which are widely investigated and presented into Table 1.

Segmented polyurethanes contain flexible and rigid chains alternately in the polymer back bone. The precursor of soft flexible segments is polymeric macromolecules with hydroxyl-end groups, while rigid segments are obtained because of reaction diisocyanate with low-molecular diol or diamine. Due to the chemical incompatibility between the two types of segments (soft and hard), segmented polyurethanes are phase separated on a nanoscale. This phenomenon leads to the creation hard and soft domains, in which hard domains (HS) are rich in rigid segments and soft domains (SS) are rich in flexible chains. Hard domains are responsible for mechanical strength and hardness, and on the other hand, soft domains provide elasticity [53]. Furthermore, hard domains act as a physical cross-linking by the so-called reversible cross-links through hydrogen bonding interactions. Therefore, these materials are excellent thermoplastic elastomers (TPUs), which allow them to be processed using thermoplastic machinery such as extrusion and injection molding for techniques. The soft segments provide elastic properties to behave like a rubber at ambient conditions.

The aim of this study is to explore the influence of graphene-like nano structured carbon fillers, RGO (reduced graphene oxide), and GNP (graphene nanoplatelets) on the morphology and proprieties of segmented polyurethane thermoplastic elastomers. The filler dispersion, filler orientation and filler-filler networking (Payne effect) have been carefully studied and compared. Morphology-property correlation has been established. Techniques such as high-resolution transmission electron microscopy (HRTEM), DSC, TGA, XRD, Fourier Transformation Infrared Spectroscopy (FTIR), and UTM have been employed to characterize the morphology and proprieties of the system. Theoretical models have been used to fit the experimental data.

## 2. Experimental Section

### 2.1. Chemicals and Materials

In the following research graphite flakes, Micro 850 (99% at. C, Asbury Carbons, Asbury, NJ, USA) was used as a precursor to obtain Graphite Oxide (GO). Also, potassium permanganate (KMnO_4_, Stanlab, >99%), hydrogen peroxide (H_2_O_2_, 30% aq.), hydrochloric acid (H_2_SO_4_, 35–38% aq.), phosphoric acid (H_3_PO_4_, 85%), sulfuric acid (H_2_SO_4_, 96%), ethanol (C_2_H_5_OH, 96%), and diethyl ether ((C_2_H_5_)_2_O), all from POCh S.A., Poland, were used in GO synthesis. Compressed argon (Ar, >99% mol.) was used in thermal reduction of GO to reduced graphene oxide (RGO). Graphene platelets (GNP) were supplied by ACS Materials (Advanced Chemicals Supplier, Medford, OR, USA). Thermoplastic polyester urethane (TPU) 52DE55 (Applicazioni Plastische Industriali S.P.A, Mussolente, Italy) was used as a nanocomposite polymer matrix. *N*,*N*-Dimethylmethanamide (DMF) ((HCON(CH_3_)_2_, 99%, POCh S.A., Gliwice, Poland) was used as a solvent in composite preparation.

### 2.2. Preparation of RGO

Reduced graphene oxide (RGO) was obtained in two-step synthesis. On the first step, graphite oxide (GO) was obtained using modified Hummer’s method described in the following article [54]. On the second step, GO was thermally reduced to RGO at a temperature of 200 °C and normal pressure for 15 min within argon. The efficiency of reduction was 30%.

### 2.3. Nanocomposites Preparation

Nanocomposites were prepared by the solvent blending method. TPU was dissolved by adding it to DMF at temperature of 80 °C with continuously stirring, the final concentration of TPU/DMF solutions was 25% by weight. Next, the proper amounts of nanofillers was added to prepared solution and was homogenized by high-shear mixer (Omni Macro Homogenizer, 35 mm mixing head diameter) with 2500 rpm for 30 min. Obtained polymer nanocomposite solutions, with good quality of dispersion, were poured onto Petri pans and the DMF was fully evaporated at 70 °C for 72 h. The obtained nanocomposites were compression molded (under a pressure of 1 ton) to thin film with 0.7 mm thickness at the temperature of 195 °C. Press molded films (Table 2) was used later for characterization.

## 3. Characterization

### 3.1. FTIR

Fourier Transformation Infrared Spectroscopy of polyurethane materials has been carried using ThermoElectron Corporation (Waltham, MA, USA) Nicolet 8700 Spectrometer in the Attenuated Total Reflectance (ATR, Gold State II) mode in the range of 500–4000 cm^−1^. The measurement was carried in standard conditions (normal pressure and at room temperature).

### 3.2. XRD

Wide-angle X-ray scattering measurements of nanofillers and polyurethane composites were performed using a Bragg–Brentano X’PERT PHILIPS diffractometer (Malvern Panalytical B. V., Almelo, Netherlands), equipped with a Cu Anode X-ray tube and diffracted beam monochromator (40 kV, 30 mA, λ Cu Kα  =  0.1542 nm). TPU samples were scanned in 2θ range from 5° to 35°.

### 3.3. TEM

Microscopy analysis (for nanofiller GNP and RGO) was performed by means of STEM-EDX technique using Transmission Electron Microscope FEI Europe (Eindhoven, The Netherlands), Tecnai F20 X-Twin coupled with EDX Spectrometer (Tecnai, Hillsboro, Oregon, USA) (samples were cut in cryo-mode using ultramicrotome). HRTEM (for nanocomposite systems) is from JEOL (Tokyo, Japan), model JEM-2100. The samples were sliced with LEICA EM UC7 Ultra Microtome with cryo-mode (EM FC7, LEICA, Wiesral, Germany) using glass knife. The thickness of the samples was 150 nm and the slices were deposited on a 200 mesh Cu Grid.

### 3.4. TG

Thermogravimetry (TG; (International Organization for Standardization) ISO 11358) of obtained materials was conducted by Netzsch TG209F3 TG analyzer (NETZSCH, Selb, Germany). Degradation process has been performed in the temperature range from 35 °C to 700 °C, nitrogen atmosphere with gas flow rate 40 mL/min, and heating rate 20 °C/min.

### 3.5. DSC

Differential Scanning Calorimetry (DSC; ISO 11357-1, 11357-3) measurements have been carried by Netzsch DSC209F1 instrument (NETZSCH, Selb, Germany) in the temperature range from −85 °C to 250 °C. The samples weight was about 10 mg, and analysis was conducted in nitrogen atmosphere with gas flow 40 mL/min. The samples were pre-heated (1st-heating) to eliminate thermal history, then cooling and 2nd-heating at rate 10 °C/min.

### 3.6. Mechanical Properties (Dynamic and Static Mode)

Dynamical Mechanical Analysis (DMA; (American Society for Testing and Materials) ASTM D4065, D7028-07) has been carried by TA Instruments DMA Q800 analyzer (DMA, West Kentucky, PA, USA). Temperature investigation mode has been performed in uniaxial tension mode, in the temperature range −100–100 °C, 1Hz frequency amplitude of deformation 20 µm and heating rate 4 °C/min using liquid nitrogen as cooling medium. Strain sweep analysis for Payne effect investigation has been conducted in three different temperatures (35 °C, 55 °C, and 75 °C), in the same tension mode and frequency of deformation, but in the amplitude range from 0.25 to 170 µm. The dimensions of thin samples were 5.0 × 5.0 × 0.7 mm^3^. Tensile (in static mode) tests were carried out using universal testing machine (UTM) Zwick/Roell Z020 (Zwick, Kennesaw, GA, USA). All of the tests were performed according to ISO 527-1 standard.

## 4. Results and Discussions

### 4.1. Characterization of Graphene Nanofillers

**Figure 1:**
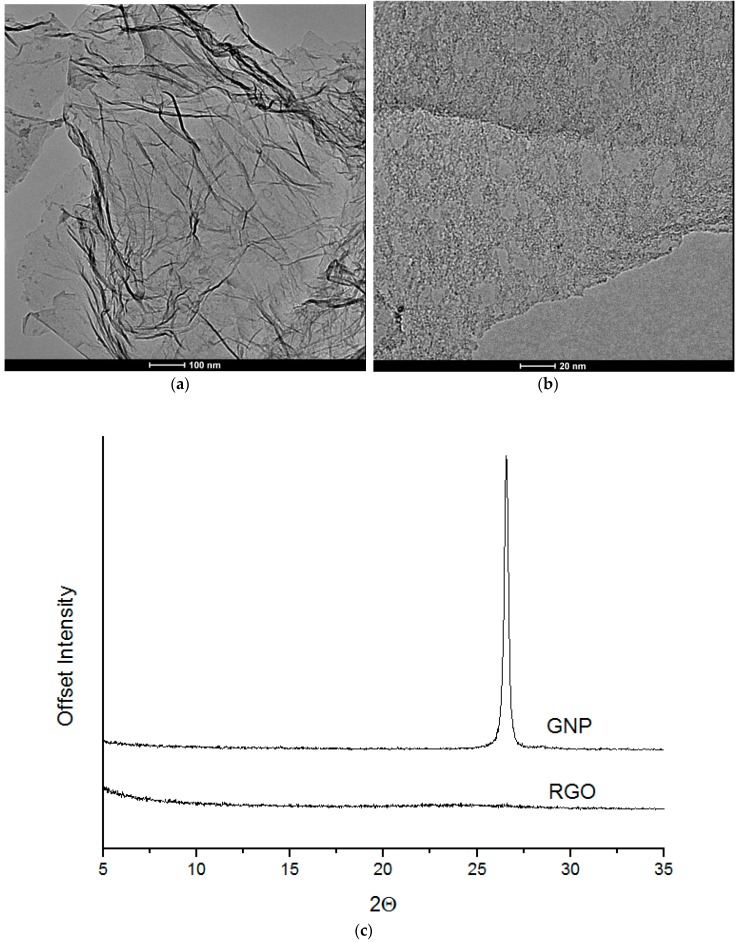
High resolution Transmission Electron Microscopy (TEM) images of graphene oxide (RGO) (**a**) and graphene nanoplatelets (GNP) (**b**) monolayers and Wide-angle X-ray scattering (WAXS) patterns of RGO and GNP nanofillers (**c**).

Prepared reduced graphene oxide (RGO) nanofiller possess good exfoliated structures (Figure 1a). Our previous study [55] confirmed that RGO nanofiller consists of about six layers (2 nm height) in a stacking nanostructures. The other nanofiller–graphene nanoplatelets (GNP) possess many defects, as observed in the form of holes within the structure (Figure 1b). GNP are seen as thin flakes with diameters between 2 and 10 nm in agreement with our previous presented study [56]. The X-ray diffraction analysis was performed on the GNP nanofiller and is characterized by the presence of diffraction maximum at 2θ = 27° (Figure 1c) in agreement with the literature report [57]. For RGO nanofiller, we have observed the absence of diffraction maximum, which could be correlated with disordered structure of this material (Figure 1c).

### 4.2. Characterization of Nanocomposites

#### 4.2.1. FTIR Spectroscopy

**Figure 2:**
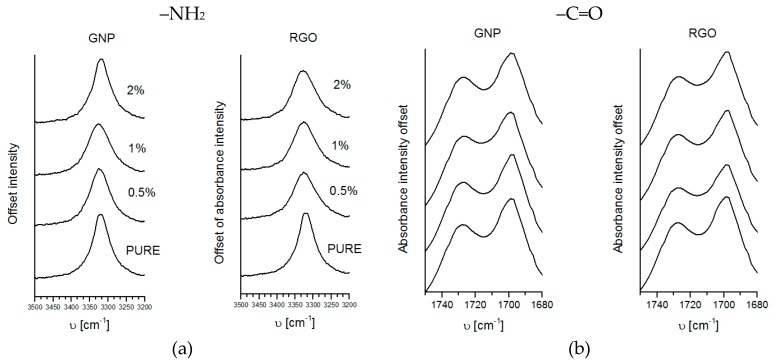
Fourier Transformation Infrared Spectroscopy (FTIR) spectra’s comparison for pure TPU (Thermoplastic polyester urethane) and TPU nanocomposites (**a**) –NH^2^ band and (**b**) –C=O band.

Based on the FTIR spectroscopy, we have the interaction between nanofiller and PU segments (soft and hard). The spectra of TPU and modified nanocomposite systems (Figure 2) are similar for both modifications GNP and RGO. The weak absorption peaks, as observed at 3319 cm^−1^, were assigned to stretching vibrations of N–H groups that were present in both types of TPU spectra. The wide base of the identified peak of NH stretching may be related to the presence of hydrogen bonds in the TPU and GNP or RGO modified TPU structure. The very strong carbonyl stretching, in the case of obtained TPUs and nanocomposite systems, appeared at 1726 cm^−1^. The FTIR analysis confirmed that the performed synthesis lead to the generation of obtain TPUs [58].

#### 4.2.2. Wide Angle X-ray Diffraction (WAXD)

**Figure 3:**
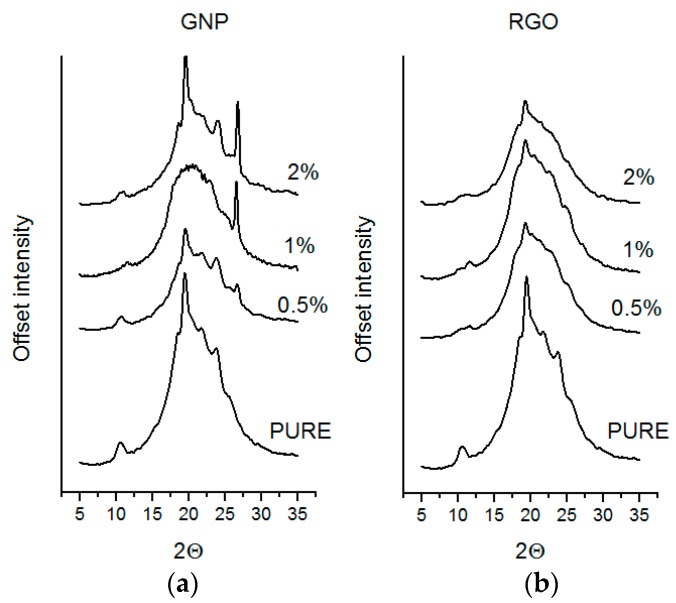
Diffraction maxima for nanocomposites (**a**) polyurethane (PU)-GNP and (**b**) PU-RGO.

Polyurethane matrix (non-modified material) possess good define, long-range ordering, crystalline structure. What is more, these materials exhibit a micro-heterophase structure, which is not visible as diffraction maxima measure using X-ray diffraction. These observations were also confirmed, based on DSC results (see. Section DSC), where soft domain (SS) crystallization and melting point are not visible. What is more, hard domain (HS) connections and bigger agglomerations cause visible diffractogram maximum at 20° (Figure 3). It was visible hard segment (HS) orientation with the addition of nanofiller into polyurethane matrix. Based on XRD diffraction maxima, lower crystalline phase content is observed at about 20°. This is connected with a lower area of segment interaction with increasing nanofiller content. Introduced nanofillers (RGO or GNP) interact with soft and hard segments to improve the direction of the HS, better separation of these domains and crystallization. For RGO based nanocomposites is visible better ordered structures in comparison to the GNP one. Especially at 17–25° and 10° maxima where TPU/GNP nanocomposites possess lower intensity, but maxima are still separated. These behaviors could be connected with different size of nanofiller (higher aspect ratio RGO over GNP), what cause block macromolecules and decrease the rotation and crystal growing direction of hard domain. Second explanation can be connected with higher tendency to infect of hard domain by RGO nanofiller and GNP one reviles in soft domains. What is more, in hard domains, more RGO is observed than for GNP nanocomposite and this present nanofiller influent on selective improvement. Otherworld’s nanofiller infect crystallinity of hard domains and block ability to recrystallization. Diffraction curves (Figure 3) show visible maxima of GNP nanofiller what can be related to difficult exfoliation process.

#### 4.2.3. Transmission Electron Microscopy (TEM)

**Figure 4:**
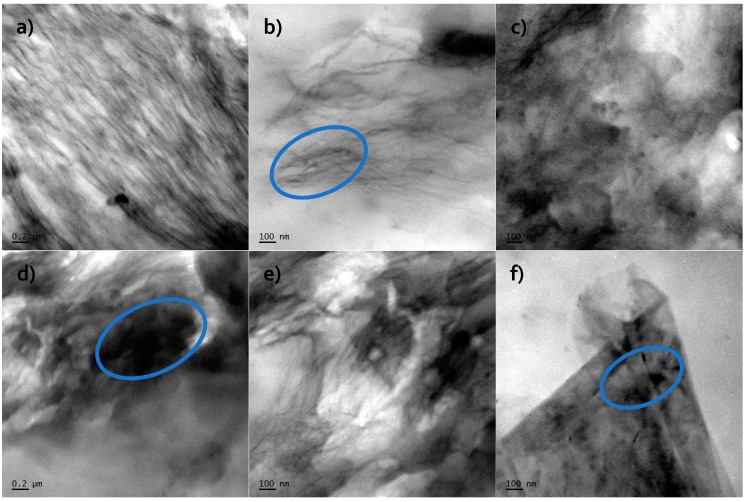
TEM microphotographs of (**a**) 1.0 TPU/RGO; (**b**) 1.0 TPU/RGO; (**c**) 2.0 TPU/RGO; (**d**) 1.0 TPU/GNP; (**e**) 1.0 TPU/GNP; and, (**f**) 2.0 TPU/GNP.

Based on Transmission Electron Microscopy (TEM) analysis, it was confirmed presents of the nanofiller (RGO or GNP) into polyurethane matrices. This is very important factor to characterize morphology of the nanofiller (size, dispersion) on the nanocomposite systems. Reduced graphene oxide (RGO) and graphene nanoplatelets (Figure 4) have layered structure to e.g., montmorillonite systems [59].

Long, dark lines represent the characteristic geometry of graphene nanofillers, which are dispersed into PU materials [60]. For TPU/RGO systems, the good dispersion (few layers-thick reduced graphene oxide sheets) obtained what clearly show in the Figure 4a–c. For TPU/GNP systems (Figure 4d–f) the larger agglomerates were observed (about 200 nm length) in comparison to the RGO filled systems. RGO nanofiller possess better compatibility to the polyurethane matrix (what relates to –O– or –OH group on the surface of the nanofiller), and therefore higher dispersion is observed. Classic polymer nanocomposite systems (filled with layer structured nanofillers) can possess intercalated or exfoliated structured [61]. Compare TEM analysis for obtained materials and XRD results (absent of diffraction maxima; describe in Section 4.2.2), exfoliated nanocomposite morphology was confirmed.

#### 4.2.4. Thermogravimetric Analysis (TG)

Segmented polyurethanes based on microheterophase structure possess different degradation temperature of hard (HS) and soft (SS) segments. Generally, hard segments degradate at lower temperatures in comparison to the degradation of soft domains of polyurethane materials. This characteristic was registered on DTG thermograms: two maximum peaks for maximum degradation temperatures for HS and SS. At degradation temperature rage, partially mixing (above 120 °C) of hard and soft domains could be possible. What is more, one maximum on DTG curve determines absent of phase separation of the PU segments.

Based on TG analysis, it was possible to verify phase separation in polyurethane system that was caused by nanofiller. Graphene based nanofiller can thermally protect the polyurethane matrix, especially at local regions. It was found for obtained nanocomposites, that high thermal conductivity of graphene, allows for effective heat transfer, causing a reduction of over-heating of hard segment domains, what is visible as higher degradation temperature of filled materials. Other possible mechanism of better thermal stability of the systems is based on lower phase separation of soft (SS) and hard segments (HS), and higher mobility of soft segments and easy transfer (dissipation) heat into polyurethane matrix. Addition of nanofiller, which reduce SS and HS mobility influent on phase separation of these systems.

**Figure 5:**
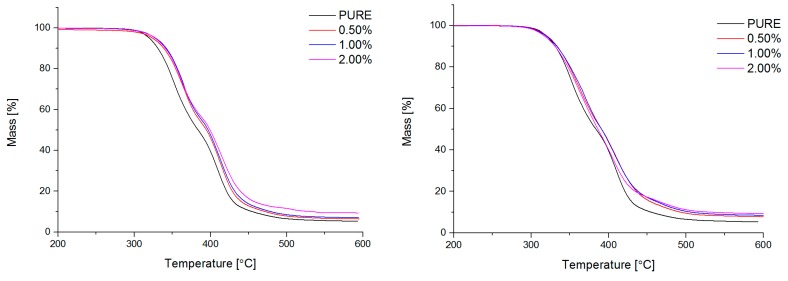

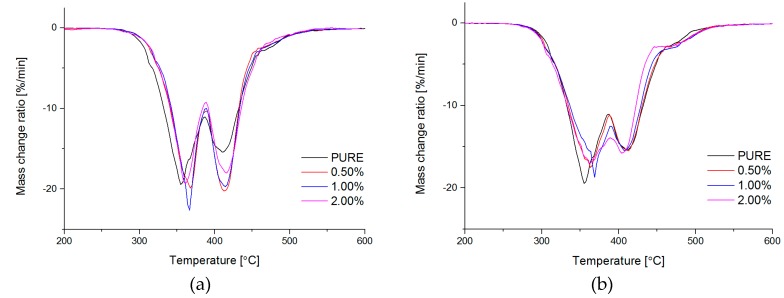
Thermogravimetry (TG) curves %mass vs. Temperature and DTG vs. temperature for TPU and TPU RGO (**a**)/GNP (**b**) nanofiller systems.

The degradation temperature for PURE TPU starts at 328 °C (T_onset_) (Figure 5). Decomposition temperature (T_onset_) shifts to 5–6 °C higher values, for RGO based nanocomposites, and 8–10 °C for TPU/GNP systems (Table 3). For obtained systems, the maximum of degradation temperature (T_DTG-HS_) shifts to the higher temperatures for modified systems (max. 12 °C for 0.5% RGO and 1.0% GNP-TPU filled nanocomposites). The degradation temperature of soft segments (T_DTG-SS_) for all of the modified materials is similar to the degradation temperature of the PURE TPU system (Table 3). It is also visible as an increasing in mass residue with addition of nanofiller to polyurethane nanocomposites (Table 3).

#### 4.2.5. Differential Scanning Calorimetry (DSC)

**Figure 6:**
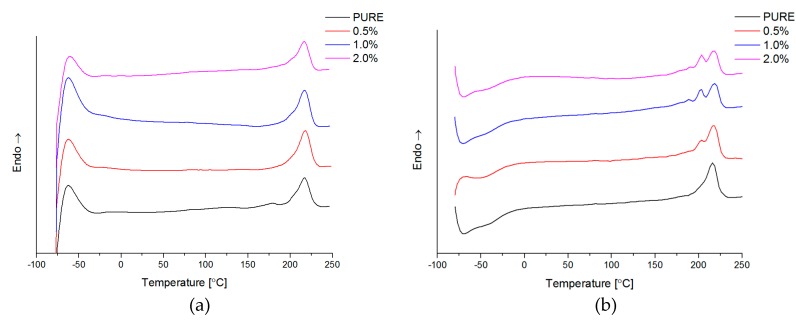
Thermograms for nanocomposites RGO (**a**); GNP (**b**) (second heating) and PURE TPU system.

**Figure 7:**
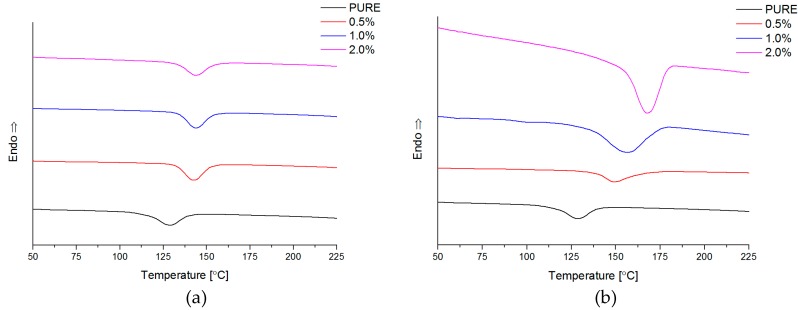
Thermograms for nanocomposites RGO (**a**); GNP (**b**) (cooling) and PURE TPU system.

For many group of polymers, which possess semicrystalline structure, the incorporation of nanofiller causes changes in the crystallization degree, size of the crystalline aggregates, and changes in crystallization rate and in the maximum crystallization temperature (T_m(max)_) observed on DSC thermograms [59]. What is more, GNP nanofiller promotes the crystallization process into polyurethane matrix what was investigated using other polymer matrices e.g., polyamide 6 [62]. Additionally, the presence of nanofiller may also affect the rate of crystallization [61]. It was noticed that higher crystallization temperatures (T_max_ ≈ 14–40 °C) (Table 4) were registered for nanocomposites containing GNP and RGO nanofiller. The addition of nanofiller causes different curve character for melting point peaks. For PURE TPU material is visible peak at 212–216 °C connected with melting point of crystalline reign from hard segments (HS). In the DSC curve, close to the 0 °C, it is a visible characteristic step that is connected with glass transition temperature (T_g_). This parameter was calculated and described, based on DMA study (Section 4.2.6) [T_g_ ≈ −27 °C (for PURE TPU) to −31 °C (for 2.0 TPU/GNP)]. For PURE TPU polyurethane matrix, DSC curve indicates that there are no crystalline regions within the soft domain, while at 216 °C there is a maximum corresponding to the melting of crystalline hard domains (T_m_). Thus, the polyurethane matrix is composed of two amorphous regions, which are arranged in rigid segments consisting of hard domains. Polyurethane matrix possesses soft and hard segments what was also confirmed using XRD investigation and correlate with DSC results. The visibility of diffraction maxima’s in XRD studies (Section 4.2.2) indicate that the ordering of polymer chains is long-range, indicating that the contents of the rigid segments in the matrix are above 25%-the hard domain (HS), resulting in larger crystalline agglomerates. GNP nanofiller add into polyurethane matrix influent on heating thermograms (Figure 6), where multi maximum peaks are visible in the temperature range 190–220 °C. Based on melting theory [14,61], the maximum of the peak at lower temperature relates to smallest crystalline areas and for larger crystalline form the temperature is higher. The effect of nanofillers (GNP and RGO) on crystallization of hard domains (HS) has also been investigated. The influence of particles on the crystallization process is clearly visible (Figure 7). The DSC curves of the TPU/GNP composites show an increase in the crystallization temperature (T_c_), which is related to the GNP activity as nucleation process in the heterocrystallization by increasing its temperature. In the TPU/RGO nanocomposites, the crystallization temperatures have practically remained unchanged, indicating its less influential factor.

#### 4.2.6. Dynamic Mechanical Analysis (DMA) and Mechanical Properties

**Figure 8:**
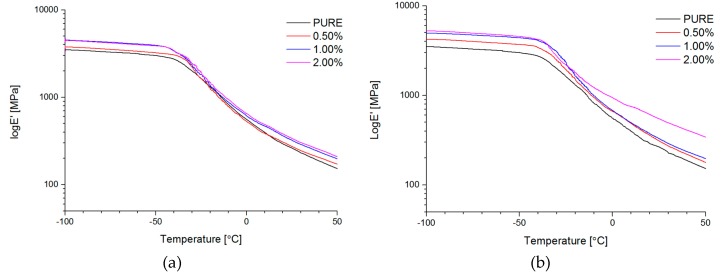
Storage modulus (E′) vs. temperature for TPU and TPU RGO (**a**)/GNP (**b**) nanofiller systems.

**Figure 9:**
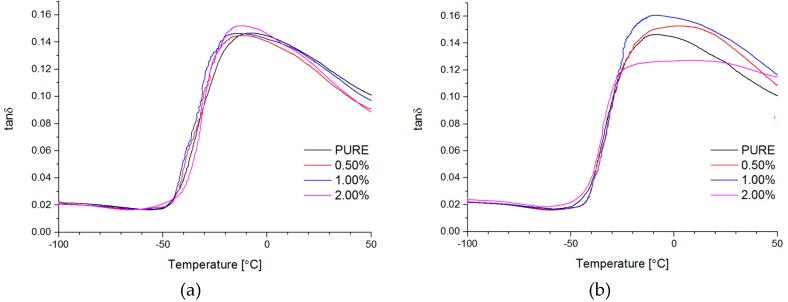
Loss tangent (tan δ) vs. temperature for TPU and TPU RGO (**a**)/GNP (**b**) nanofiller systems.

**Figure 10:**
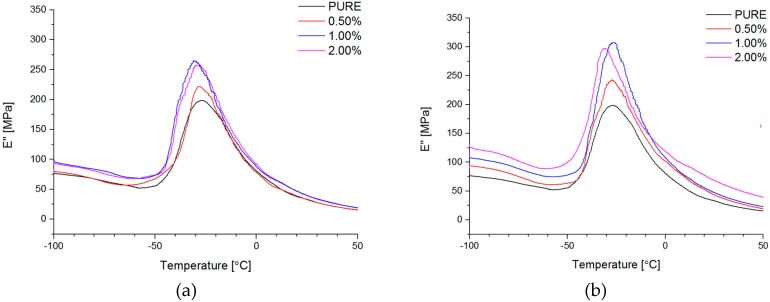
Loss modulus (E″) vs. temperature for TPU and TPU RGO (**a**)/GNP (**b**) nanofiller systems.

Dynamic mechanical properties were analyzed for all of the obtained materials. Based on curve behavior, it was visible high hard segments (HS) content for non-modified material (PURE TPU) (Figure 8, Figure 9 and Figure 10). For modified systems (RGO and GNP) with increasing nanofiller content, higher values of E′ were obtained (Figure 8, Table 5) in a wide range of temperatures.

Interesting information can derive from tan δ versus temperature at the T_g_ region. There is only a slight change of T_g_ value of TPU nanocomposite in comparison to the PURE TPU (Figure 9). Tangent δ peak values slightly decrease (from 0.15 to 0.12), especially for 2 wt % GNP, which can be related to less damping behavior of GNP filled TPU [63].

Mechanical properties of polyurethane nanocomposite materials, in the static mode, were presented in the Table 5. With an increasing amount of nanofiller (both RGO and GNP), the tensile strength of modified materials is higher in comparison to the pure TPU matrix. Addition of 2 wt % RGO into polyurethane causes, higher values of tensile strength [*δ_max_*] about 30% and equal 44%, for GNP nanofiller and for RGO modification, respectively. RGO nanofiller enhanced mechanical properties of polyurethane matrix more in comparison to the GNP one. This is due to the presence of functional group on the surface of the RGO nanofiller, which relates to an increased compatibility to the matrix. These results correspond to the Santosh Kumar Yadav et al. studies [44]. The authors also confirmed that the best mechanical properties for polyurethane nanocomposites, containing functionalized graphene nanoplatelets (f-GNP), are registered for the same amount of the nanofiller in the matrix.

#### 4.2.7. Payne Effect Analysis

**Figure 11:**
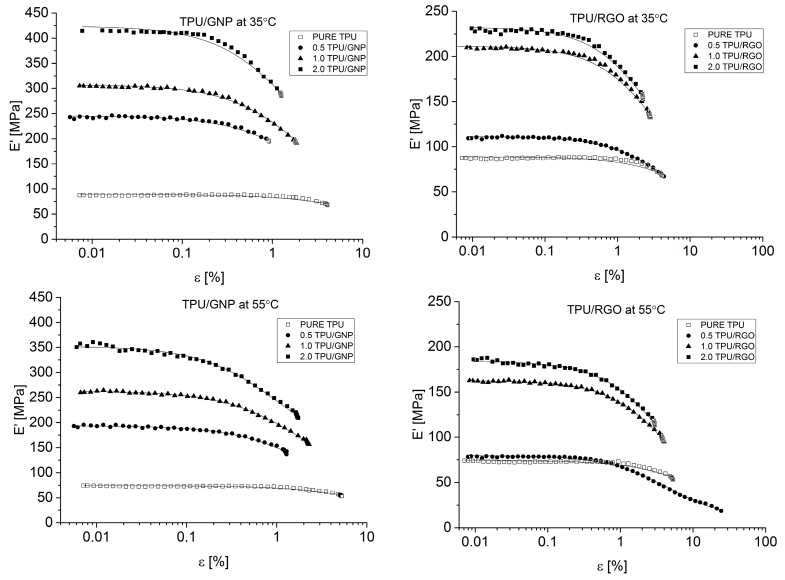

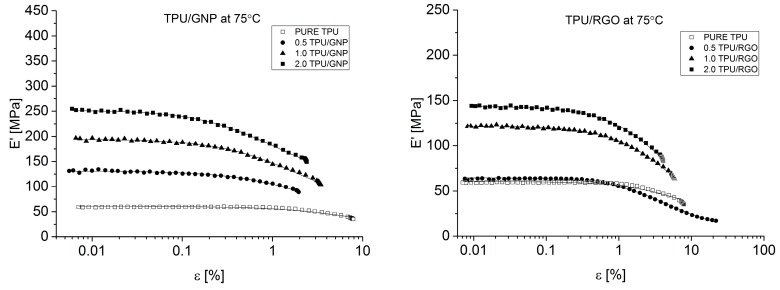
Payne effect comparison based on storage modulus (E′/MPa) vs. strain (ε/%) changes.

The results of dynamic strain scan analysis have shown that the storage modulus (E′/MPa) of nanocomposite systems increased with nanofiller (RGO or GNP) loadings (Figure 11, Table 5), and also show a similar trend for different nanofiller content. The unfilled polyurethane matrix does not display significant changes in storage modulus with strain amplitude (Figure 11). Introducing nanofiller into the PU component causes an increase in storage modulus, which is especially visible for samples with 1 wt % and 2 wt % (GNP and RGO), where these amount of nanofiller give higher values of E′ and visible drop with the strain amplitude (Table 5, Table 6 and Table 7). For the same temperature investigation study, the highest value of E′ possessed for nanocomposite system stain 2 wt % of GNP modificator (416 MPa for 2.0 TPU/GNP, in comparison to the PURE TPU storage modulus equal 87 MPa, Table 7). With an increasing temperature (35 °C, 55 °C, 75 °C), the storage modulus (E′) possesses is lower for unfilled and filled materials (for both used nanofillers). It was observed that in all nanocomposite materials, the amplitude of the Payne effect decreases with an increasing temperature, which is connected with the weakening of filler-filler interactions [64]. Payne effect is widely characterized as filler dispersion and filler network in elastomeric polymer matrix [65]. This effect is described as changes of storage modulus (E′/MPa) filled materials at small strain amplitude, as compared to the non-filled polymeric materials. Strain amplitude sensitivity is considered as a typical non-viscoelastic response in the filled materials. The reduction of storage modulus (E′) with strain (ε/%), increasing amplitude is also characteristic for composite or nanocomposite materials [66]. Payne effect is also connected to the formation of filler network by filler-filler and filler-matrix interactions [65].

## 5. Conclusions

Based on this study it was successfully obtained polyurethane nanocomposites containing reduced graphene oxide (RGO) or graphene nanoplatelets (GNP). The addition of RGO or GNP undoubtedly influenced investigated properties of the polyurethane matrix.

The authors showed the interaction behavior of the nanofillers with the polyurethane matrix by using spectroscopic and diffraction investigation. Morphology investigation confirmed good exfoliation nanoparticles into the polyurethane matrix and shows a better compatibly of RGO nanofiller to the matrix, in comparison to the GNP (where higher agglomeration of this nanofiller is observed, Figure 4d). Phase transition investigation gives information about the effect on melting and crystallization behavior, where GNP nanofiller causes an increased crystallization temperature of the nanocomposite systems. The storage modulus (E′) of nanocomposites containing TPU/GNP materials significantly increased in comparison to the non-modified (PURE TPU) material. The modulus and thermal stability of the TPU/GNP (RGO) nanocomposites at 2 wt % nanofiller loading are higher, especially above glass transition temperature (T_g_), respectively, than those of the PURE TPU material. It is seen (Table 5; *δ_max_*) that the addition of the nanofillers improves the mechanical strength of TPU matrix. Enhanced mechanical properties were observed when add maximum 2 wt % of nanofiller and higher tensile strength properties obtain for RGO nanofiller in comparison to the GNP modificator.

Both nanofillers, GNP and RGO, enhanced the mechanical and thermal properties of the TPU material. Furthermore, it should be highlighted that by using RGO and GNP nanofiller, the Payne effect is visible for obtained nanocomposite systems. It is demonstrated that different Payne effect behavior is visible for commercial graphene nanoplatelets (GNP) and synthesized reduced graphene oxide (RGO) nanofiller. We could observe agglomeration (strong filler/filler interaction) at higher filler loading, which was evidenced by the high Payne effect. When comparing with results in literature [67] based on TPU matrix and the same amount of filler, nanocomposites with a good balance between thermal and mechanical properties were achieved in this study. The authors believe that these industries based materials could be easily applied in many fields, due to their enhanced properties.

## Figures and Tables

**Table 1:** Comparison of the improvements by the nanofilles on the polyurethane matrices.

Polyurethane Matrix	Nanofiller	Content	Processing Method	Tensile Strength	Thermal Stability	Important Improvement
TPU [45]	OMMT	1.0 wt %	melt	Increased 7 to 27 MPa	-	E′ increased
TPU [46]	OMMT	5.0 wt %	melt	Increased 488 to 908 KPa (RPA)	-	G′ increased, 5 wt % RPT
TPU [47]	OMMT	1.0 wt %	in situ	Increased 32 to 47 MPa	Increased 10 °C	T_m_ increased
WBPU [48]	A-CNT	1.5 wt %	in situ	Increased 7 to 9 MPa	-	54 to 62 hardness
PU [49]	SWNT	2.0 wt %	in situ	Increased 6 to 9 MPa	Not changed	T_g_ decreased
TPU [50]	MWCNT	2.0 wt %	melt	Increased 2 to 3 MPa	Increased 13 °C	Increased modulus
TPU [19]	TRG/iGO	1.6 vol %	solution	E′ increased	-	×10 tensile stiffness
TPU [51]	GNPs	2.7 vol %	solution	decreased	-	-
PU [44]	f-GNP	1.5 wt %	solution	Increased 17 to 23 MPa	Increased 30 °C	Enhanced shape memory
TPU [43]	RGO	0.1 wt %	solution	Increased 8 to 29 MPa	Increased 6 °C	410% toughness 8% hardness
PU [52]	GO	1.0 wt %	solution	Increased 40%	-	280% Young modulus (3 wt %)
PU [14]	GO	4.0 wt %	solution	Increased 5 to 27 MPa	-	×4.8 Young modulus
PU [32]	GNS	2.0 wt %	in situ	Increased 11 to 36 MPa	Increased 40 °C	202% storage modulus

Abbreviations: TPU—thermoplastic polyurethane; OMMT—organically modified montmorillonite; E′—storage modulus; RPA—rubber process analyzer; G’—dynamic storage modulus; RPT—rheological percolation threshold; T_m_—melting temperature; WBPU—waterborne polyurethane; A-CNT—acid treated carbon nanotube; SWNT—single-walled carbon nanotube; PU—polyurethane; T_g_—glass transition temperature; MWCNT—multiwall carbon nanotube; TRG—thermally reduced graphene oxide; iGO—isocyanate treated GO; GNPs—graphene nanoplatelets; f-GNP—functionalized graphene nanoplatelets; GO—graphene oxide; RGO—reduced graphene oxide; GNS—graphene nano-sheets.

**Table 2:** Samples designation and nanofillers content.

Samples	Nanofiller Content/wt %
PURE TPU	0%
0.5 TPU/GNP	0.5%
1.0 TPU/GNP	1.0%
2.0 TPU/GNP	2.0%
0.5 TPU/RGO	0.5%
1.0 TPU/RGO	1.0%
2.0 TPU/RGO	2.0%

**Table 3:** Thermogravimetric results for TPU (Thermoplastic polyester urethane) and TPU RGO/GNP nanofiller systems.

Composite	T_onset_ (°C)	T_95%_ (°C)	T_90%_ (°C)	T_50%_ (°C)	T_DTG-HS_ (°C)	V_DTG-HS_ (%/Min)	T_DTG-SS_ (°C)	V_DTG-SS_ (%/Min)	Remains (% Mass)
PURE TPU	328	321	333	387	356	−19.5	411	−15.5	5.5
0.5 TPU/RGO	333	324	339	395	368	−19.9	413	−20.2	6.5
1.0 TPU/RGO	333	328	342	397	366	−22.8	415	−19.7	7.0
2.0 TPU/RGO	334	327	341	399	361	−19.3	416	−18.0	9.4
0.5 TPU/GNP	326	320	333	391	364	−17.7	413	−15.5	7.5
1.0 TPU/GNP	323	319	333	391	368	−18.7	408	−15.5	8.2
2.0 TPU/GNP	328	317	331	386	362	−17.1	404	−15.8	9.0

**Table 4:** Differential Scanning Calorimetry (DSC) parameters, T_m_–melting temperature, ∆H_m_–enthalpy of melting, T_c_–crystallization temperature, ∆H_c_–enthalpy of crystallization for TPU (PURE), and TPU RGO/GNP nanofiller systems.

Materials	T_m_ (Max) (°C)	∆H_m_ (J/g)	T_c_ (Max) (°C)	∆H_c_ (J/g)
PURE TPU	216	11.9	129	−18.2
0.5 TPU/GNP	203; 217	11.9	149	−18.7
1.0 TPU/GNP	189; 203; 219	12.5	157	−18.6
2.0 TPU/GNP	191; 204; 218	12.8	169	−16.3
0.5 TPU/RGO	219	17.8	143	−17.8
1.0 TPU/RGO	200; 218	15.6	144	−16.6
2.0 TPU/RGO	192; 201; 217	11.4	144	−17.1

**Table 5:** Dynamic Mechanical Analysis (DMA) results for TPU and TPU RGO/GNP nanofiller systems.

Composites	E′_−70_ (GPa)	E′_−35_ (GPa)	E′_25_ (GPa)	T_g_ (°C)/E″	δ_max_ (MPa)
PURE TPU	3.2	2.4	0.3	−27	30.4 ± 1.5
0.5 TPU/GNP	3.9	3.0	0.3	−27	32.7 ± 1.6
1.0 TPU/GNP	3.0	2.3	0.2	−26	36.8 ± 1.8
2.0 TPU/GNP	4.9	3.7	0.5	−31	39.1 ± 2.0
0.5 TPU/RGO	3.5	2.8	0.3	−28	37.1 ± 1.9
1.0 TPU/RGO	4.2	2.9	0.3	−30	39.5 ± 2.0
2.0 TPU/RGO	4.1	3.0	0.3	−29	44.0 ± 2.2

**Table 6:** DMA (Dynamical Mechanical Analysis) results for TPU and TPU RGO/GNP nanofiller systems.

Nanofiller Content [% of Weight]	E′_rel_ 35 °C		E′_rel_ 25 °C	
	GNP	RGO	GNP	RGO
0.5	1.25	1.17	1.17	1.04
1.0	0.97	1.20	0.68	1.21
2.0	1.54	1.24	2.07	1.27

**Table 7:** Payne effect comparison based on storage modulus changes.

Composites	35 °C			55 °C			75 °C		
	E_0_ (MPa)	E_∞_ (MPa)	∆E (MPa)	E_0_ (MPa)	E_∞_ (MPa)	∆E (MPa)	E_0_ (MPa)	E_∞_ (MPa)	∆E (MPa)
PURE TPU	87	87	-	74	74	-	59	59	-
0.5 TPU/GNP	240	197	43	193	154	39	134	106	28
1.0 TPU/GNP	304	228	76	263	194	69	196	144	52
2.0 TPU/GNP	416	314	102	358	241	117	248	181	67
0.5 TPU/RGO	110	98	12	79	69	10	64	55	9
1.0 TPU/RGO	209	179	30	162	136	26	121	106	15
2.0 TPU/RGO	231	193	38	186	150	36	144	119	25
